# Distinct Roles of *Plasmodium* Rhomboid 1 in Parasite Development and Malaria Pathogenesis

**DOI:** 10.1371/journal.ppat.1000262

**Published:** 2009-01-16

**Authors:** Prakash Srinivasan, Isabelle Coppens, Marcelo Jacobs-Lorena

**Affiliations:** Malaria Research Institute and Department of Molecular Microbiology and Immunology, Johns Hopkins School of Public Health, Baltimore, Maryland, United States of America; Weill Medical College of Cornell University, United States of America

## Abstract

Invasion of host cells by the malaria parasite involves recognition and interaction with cell-surface receptors. A wide variety of parasite surface proteins participate in this process, most of which are specific to the parasite's particular invasive form. Upon entry, the parasite has to dissociate itself from the host-cell receptors. One mechanism by which it does so is by shedding its surface ligands using specific enzymes. Rhomboid belongs to a family of serine proteases that cleave cell-surface proteins within their transmembrane domains. Here we identify and partially characterize a *Plasmodium berghei* rhomboid protease (PbROM1) that plays distinct roles during parasite development. PbROM1 localizes to the surface of sporozoites after salivary gland invasion. In blood stage merozoites, PbROM1 localizes to the apical end where proteins involved in invasion are also present. Our genetic analysis suggests that PbROM1 functions in the invasive stages of parasite development. Whereas wild-type *P. berghei* is lethal to mice, animals infected with PbROM1 null mutants clear the parasites efficiently and develop long-lasting protective immunity. The results indicate that *P. berghei* Rhomboid 1 plays a nonessential but important role during parasite development and identify rhomboid proteases as potential targets for disease control.

## Author Summary

Malaria is one of the major infectious diseases and is responsible for the death of more than a million people, mostly children under the age of five. *Plasmodium*, the causative agent of malaria, is transmitted by female *Anopheles* mosquitoes. Successful development of the parasite requires efficient recognition, attachment, and invasion of host cells. Several parasite cell-surface molecules have been implicated in these processes and may require proteolytic processing in order for the parasite to complete invasion. Rhomboid family proteins are serine proteases that cleave within the transmembrane region of their substrates. Here, we use a genetic approach to study the function of *Plasmodium berghei* rhomboid 1 (PbROM1). PbROM1 is expressed in both vertebrate and mosquito stages of parasite development, and the protein is present in secretory organelles that contain other parasite molecules required for invasion. We find that PbROM1 is required for efficient infection of both the mosquito and the vertebrate host. Interestingly, we also find that mice infected with ROM1(−) parasites clear the infection efficiently and are protected upon subsequent wild-type parasite challenge. Our study suggests a role for PbROM1 throughout parasite development and identifies ROM1 as a target for disease intervention.

## Introduction

For successful development and transmission, *Plasmodium* has to invade multiple cell types both in the mammalian host and in the mosquito vector. Much of our knowledge about the molecular mechanisms of invasion comes from the study of *P. falciparum* merozoite invasion of red blood cells (RBCs). RBC invasion involves an initial attachment followed by re-orientation and entry of the parasite into the host cell [1]. There are two main classes of parasite surface molecules, the GPI-anchored proteins such as the merozoite surface protein family (MSP) [2] and transmembrane domain-containing proteins such as AMA1 [3],[4], erythrocyte binding-like family (EBL) [5],[6] and reticulocyte binding-like family proteins (RBL) [7],[8]. A few host-cell receptors to which these ligands bind have been identified [9]–[12].

In the mosquito, motility plays an important role in ookinete and sporozoite invasion. Motile ookinetes form within the mosquito blood meal and invade the midgut epithelium. After exiting on the basal side facing the hemocoel they differentiate into sessile oocysts [13]. Subsequently, sporozoites released from mature oocysts invade the salivary glands from where they are delivered to the vertebrate host by a mosquito bite. These sporozoites travel through the blood stream until they reach the liver, where they invade and infect hepatocytes. All three invasive forms (ookinetes, sporozoites in the mosquito and sporozoites in the mammalian host) utilize the same actin-based motor for entry into the host cell. Thrombospondin-related anonymous protein (TRAP) family homologues constitute one class of protein required for motility and host cell invasion [14]–[16]. The extracellular domains of TRAP interact with host-cell receptors, while the cytoplasmic tail links to the actin-myosin cytoskeleton [17]. As the parasite glides, the parasite surface ligand-receptor complexes translocate towards the posterior end. Dissociation of these interactions by proteolytic processing is thought to be important, as this enables the parasite to move forward [18]–[20]. In another Apicomplexan parasite-*Toxoplasma*-the TRAP homologue MIC2 is cleaved within its transmembrane domain releasing the receptor-binding domain from the parasite surface [18] and *Plasmodium* merozoite TRAP (MTRP) also appears to be cleaved in a similar manner [16].

Rhomboid-family (ROM) proteins are serine proteases that cleave their substrates within their membrane domain [21],[22]. Multiple rhomboid-family proteins have been identified in the genomes of *Plasmodium* and *Toxoplasma* [23]. Cleavage requires the presence of helix-destabilizing residues within the membrane domain of substrates [24]. Indeed, Apicomplexan surface proteins such as EBL and RBL proteins, AMA1, TRAP and their homologues contain such helix-destabilizing residues [23]. Assays in cultured mammalian cells identified possible substrates for both Toxoplasma and *Plasmodium falciparum* rhomboid proteins [25],[26]. *Toxoplasma* ROM5 localizes to the posterior end of the parasite and can cleave MIC2 within its transmembrane domain [25],[27]. *Plasmodium* does not have a ROM5 homologue but ROM4 is able to cleave EBA175 [28], an EBL family protein involved in binding to erythrocytes [10]. Processing of EBA175 within its membrane domain appears to be essential for parasite invasion [28].

Here we report on experiments investigating the role of *Plasmodium berghei* rhomboid 1 (PbROM1) during parasite development in the vertebrate host and the mosquito vector. Our data suggests a role for PbROM1 throughout *Plasmodium* development and indicate a role in invasion of host cells. We also find that a null PbROM1 mutant is efficiently cleared from mice and that these animals are protected from a subsequent lethal challenge of wild-type *P. berghei*. These findings identify a unique target for interfering with both disease causing and disease transmitting forms of the parasite.

## Materials and Methods

Parasite maintenance and mosquito infections were performed as described previously [29]. We used *Anopheles stephensi* mosquitoes, *Plasmodium berghei* ANKA 2.34 parasites and female Swiss Webster mice in all our studies.

### PbROM1 antibody production and immunofluorescence assays

Antibodies were raised in rabbit against the N-terminal 52 amino acids of PbROM1 expressed in bacteria as a fusion protein using the pBAD expression system (Invitrogen). *P. berghei* schizonts, merozoites and sporozoites were fixed in ice-cold methanol and incubated for 1 h with the anti-PbROM1 antibody diluted 1∶500. Midgut and salivary gland sporozoites were obtained by gently homogenizing the infected tissues and centrifuging to remove cell debris. A anti-AMA1 monoclonal antibody (28G2) that recognizes the highly conserved cytoplasmic tail [30] was also used to label schizonts and merozoites, while a anti-CSP monoclonal antibody (3D11) that recognizes the repeat region [31] was used to label midgut and salivary gland sporozoites. Slides were then incubated for 1 h with Alexa Fluor 488-conjugated anti-rabbit IgG and rhodamine-conjugated anti-mouse or anti-rat IgG secondary antibodies. After washing, images were visualized in a Leica upright fluorescent microscope with a 100× objective and images were captured with a SPOT camera.

### Immunoelectron microscopy

Sporozoite-infected salivary glands were fixed in 4% paraformaldehyde (Electron Microscopy Sciences, PA) in 0.25 M HEPES (pH7.4) for 1 h at room temperature, followed by 8% paraformaldehyde in the same buffer overnight at 4°C. The fixed glands were permeabilized, frozen and sectioned as previously described [32]. Sections were immunolabeled with rabbit anti-PbROM1 antibodies (1∶20 in PBS/1% fish skin gelatin), then with anti-rabbit IgG, followed by 10 nm protein A-gold particles (Department of Cell Biology, Medical School, Utrecht University, the Netherlands) before examination with a Philips CM120 Electron Microscope (Eindhoven, the Netherlands) under 80 kV.

### Generation of PbROM1 disruptants

For targeted disruption of the PbROM1 gene, a disruption plasmid was constructed by PCR amplification with primers, PbROM1(−)F-5′CCATACATTAGCAGAGTATAGGGA3′ and PbROM1(−)R-5′ACTTGCAC CCACTTTTATTGTAC3′ using *P. berghei* genomic DNA as template. Cloning into the *P. berghei* transfection vector [33] resulted in plasmid pROM1. This plasmid was linearized at the unique NdeI site and transfected into *P.berghei* schizonts as described [34]. To confirm disruption of the PbROM1 gene, integration-specific PCR was performed using specific primer combinations, P1-5′CGAGCAACAATGTCTGAC3′, P2-5′GAGTTCATTTTACACAATCC3′ and P3-5′TAATACGACTCACTATAGGGAGA3′. Disruption was also confirmed by RT-PCR using primers PbROM1F-5′TTATTACGGAGTGTTTCTTC3′ and PbROM1R-5′CGGAGAAATACATAGATTA3′ *P.berghei* circumsporozoite gene primers CSF-5′GTACCATTTTAGTTGTAGCGTC3′ and CSR-5′CATCGGCAAGTAATCTGTTG3′ were used as positive control.

### Phenotypic analysis of PbROM1 disruptants

The ability of the parasites to differentiate into gametocytes and form male gametes (exflagellation) was assessed as described previously [35]. *An. stephensi* mosquitoes were fed on infected mice and the ability of the disruptant parasites to form ookinetes (24 h) and oocysts (day 15) was examined microscopically. To assess ookinete numbers, individual midguts were dissected 24 h after feeding. Ookinete numbers were calculated after examining a Giemsa-stained smeared preparation of the midgut contents and counting both ookinetes and red blood cells. We assumed that each mosquito ingested 2 µl [36] and that mouse blood has 4×10^9^ RBCs/ml [37]. Mature oocysts were counted on day 15 by direct light microscopic examination of dissected midguts. Sporozoites were isolated from midgut oocysts and salivary glands and counted on day 25–26 using a hemocytometer.

### Gliding motility assay

Sporozoites isolated from salivary glands were incubated for 15 minutes at 37°C in chamber slides coated with BSA. The supernatant was gently aspirated and sproozoite trails were fixed with 4% paraformaldehyde. The trails were visualized by labeling them with anti-CSP (mAb 3D11) antibody and rhodamine conjugated anti-mouse secondary antibody.

### TRAP processing assay

Sporozoites were isolated from salivary glands on ice and partially purified by passing through glass wool to remove mosquito debris. Protease inhibitors *N*-tosyl-L-lysine chloromethyl ketone (TLCK, 20 mM stock in water) and phenylmethylsulfonyl fluoride (PMSF 100 mM stock in ethanol) were obtained from Sigma. 30000 sporozoites were incubated at 4°C or 37°C in the presence or absence of protease inhibitors for 1h. EDTA was used to rule out nonspecific processing by metalloproteases. Parasite lysates were run on a SDS-PAGE and transferred onto PVDF membrane. These were probed with anti-TRAP antibodies that recognize the repeat region of the protein, followed by peroxidase-conjugated secondary antibody. To determine TRAP processing in PbROM1(−) parasites, 30000 wild-type and PbROM1(−)sporozoites were analyzed by SDS-PAGE as mentioned above.

### Mice infections with sporozoites

Sporozoites isolated from salivary glands were counted using a hemocytometer and mice were injected intravenously with 150, 1000 or 10000 sporozoites. Infection efficiency was assayed by monitoring the pre-patent period of blood stage infection after sporozoite injection. Prepatent period is the time elapsed between mouse infection and when the first infected red blood cell (RBC) was observed upon examination of at least 25,000 RBCs. For quantifying efficiency of liver infection, mice were injected intravenously with either 10^3^ wild-type or 10^3^ PbROM1(−) sporozoites. Animals were sacrificed 36–40 h after sporozoite injection and total RNA was prepared using Trizol reagent. *P. berghei* 18S rRNA was quantified using primers (PbrRNA1-5′TGGGAGATTGGTTTTGACGT TTATGT3′ and PbrRNA2-5′ AAGCATTAAATAAAGCGAATACATCCTTAC3′) as described [38] and the results were normalized using mouse GAPDH. Results from 4 mice per group are expressed as mean±s.d. of rRNA copy number.

### Parasite challenge

Mice were infected with either PbROM1(−) sporozoites or infected RBCs as described above. Parasitemia was checked every day until at least 30 days after the last PbROM1(−) parasites was detected. To confirm complete parasite clearance, 3×10^7^ RBCs from these animals were injected into naïve mice and these animals were observed for 30 days to ensure that no infection resulted. After complete remission, the PbROM1(−) infected mice were challenged by intravenous (iv) or intraperitoneal (ip) injection of 10^5^ wild-type *P. berghei* iRBCs. A second challenge was performed either 33 days or 7 months after the first challenge and a third 9 months after the first. Parasitemia was followed as described above. Protection is defined as the number of animals that survive the challenge.

## Results

### PbROM1 is conserved in all *Plasmodium* species

*Plasmodium berghei* ROM1 (PbROM1) was initially identified in a subtractive hybridization screen for genes expressed during parasite development in the mosquito [29]. PbROM1 encodes a protein predicted to have seven transmembrane domains carrying a conserved, membrane-embedded Asparagine, Glycine-X-Serine and Histidine “rhomboid” motif (Figure S1). At least seven rhomboid genes were identified in the genome of various *Plasmodium* species [23]. Though PbROM1 homologues are highly conserved among rodent (92% identity) and human malaria species (55% identity), sequence identity among rhomboid genes of a given species is very limited (<20%, data not shown). This points to independent evolution of different rhomboid genes and suggests that each rhomboid protein plays distinct functions in the parasite life cycle.

### PbROM1 is expressed in multiple invasive stages

**Figure 1:**
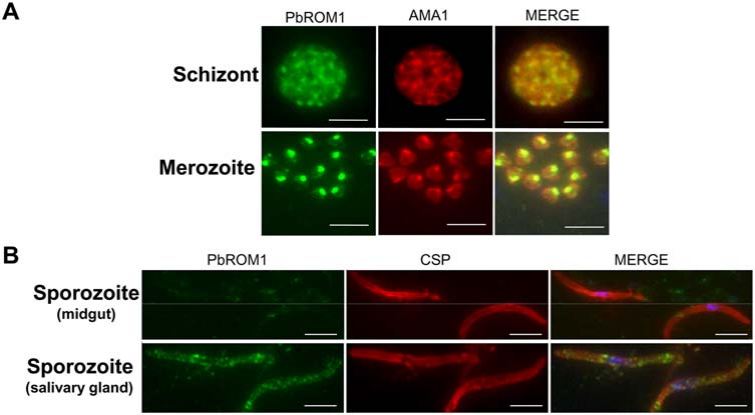
Localization of PbROM1 protein in merozoites and sporozoites. PbROM1 protein expression was assayed by indirect immunofluorescence (IFA). (A) IFA of fully segmented schizonts and free merozoites, double labeled with anti-PbROM1 (green) and anti-AMA1 (red) antibodies. (B) IFA of midgut and salivary gland sporozoites double labeled with anti-PbROM1 (green) and anti-CSP (red) antibodies. Little or no PbROM1 protein can be detected in midgut sporozoites while the protein is distributed in a punctuate pattern throughout salivary gland sporozoites. DAPI is shown in blue in the merged panels. The dotted line separates the fields of two separate images. PbROM1, *P. berghei* rhomboid 1, AMA1, apical membrane antigen 1, CSP, circumsporozoite protein. (Scale bars, 3 µm).

**Figure 2:**
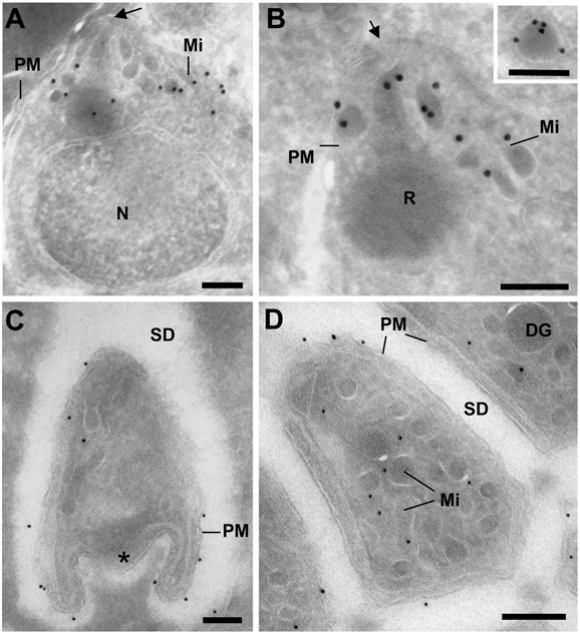
Immuno-electron microscopic localization of PbROM1 in salivary gland sporozoites. (A,B) Immunogold labeling of merozoites. PbROM1 is detected in the apical end (arrows) of merozoites within secretory organelles, predominantly within micronemes (Mi). The insert in panel B shows a microneme from another merozoite labeled with gold particles. (C,D) Immunogold labeling with anti-PbROM1 antibody of *P. berghei*-infected mosquito salivary gland sporozoite cryosections. The protein is detected on the parasite plasma membrane (PM) as well as on the membrane of micronemes (Mi) (see text for distribution statistics). The typical folded posterior end seen in sporozoites is marked with asterisk. DG: dense granule, SD: salivary duct, N: Nucleus, R: Rhoptries. (Scale bars, 250 nm).

Microarray analysis indicates that PfROM1 is expressed in both mosquito and vertebrate forms of the parasite [39]. We have produced an antibody to the first 52-amino acids of PbROM1 and used it to investigate protein expression and subcellular localization. The protein is expressed in both blood- and mosquito-stage parasites. PbROM1 protein has a punctate distribution in segmented (mature) schizonts and localizes to the apical end of free merozoites (Figure 1A). A number of organelles such as rhoptries and micronemes are found in the apical tip of the merozoite. These organelles secrete parasite proteins involved in host recognition and invasion. AMA1 (apical membrane antigen 1) is a micronemal protein required for invasion of RBCs and is also found on the surface of merozoites (Figure 1A). Immunoelectron microscopy confirmed the apical localization of PbROM1 and >85% of the gold label were found in micronemes (Figure 2A and 2B). PbROM1 expression is limited to schizonts and free merozoites and is not detectable in ring or trophozoite stages (data not shown). This is in agreement with the microarray analysis of *P. berghei* asexual stages in which PbROM1 is induced only in mature schizonts [40].

In mosquito stages, the PbROM1 transcript was initially identified among RNAs from mosquito midguts infected with mature oocysts [29]. Despite this, little or no protein was detected in sporozoites from these oocysts (Figure 1B). In contrast, PbROM1 protein is detected in sporozoites after invasion of mosquito salivary glands (Figure 1B). Immuno-electron microscopy more precisely localized PbROM1 in such sporozoites (Figure 2C and 2D). The protein is present along the entire length of the sporozoite both on the surface as well as in micronemes. We examined 65 parasite cryosections to quantify the distribution of PbROM1 in different cellular locations. Most of the gold particles were present on the sporozoite plasma membrane (76.4%) and in the micronemal membrane (17.7%) while the remaining particles were located over other parasite organelles (3.3%) and the mosquito salivary duct (2.6%).

### PbROM1 gene disruption

**Figure 3:**
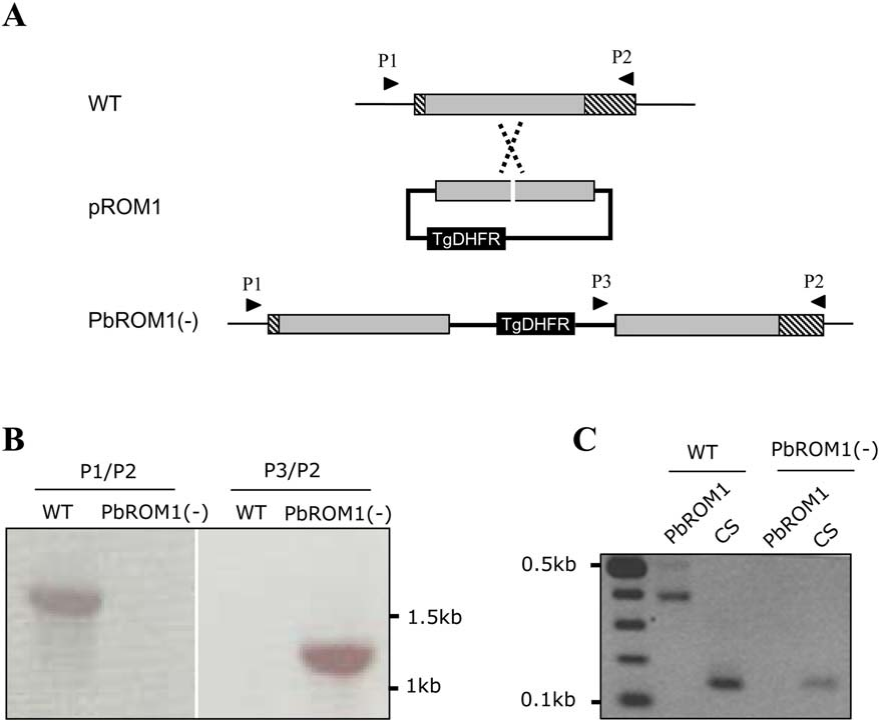
PbROM1 gene disruption. (A) *Schematic representation of the targeting strategy.* The wild-type PbROM1 genomic locus (WT) was targeted with an NdeI-linearized plasmid (pROM1) containing the 5′ and 3′ truncations of the PbROM1 open reading frame and the TgDHFR positive selection marker. Upon a single crossover event, the region of homology is duplicated, resulting in two truncated, nonexpressed PbROM1 copies in the integrated locus [PbROM1(−)]. The homologous regions in the disruption plasmid are shaded gray. Arrowheads indicate primer pairs used to confirm gene disruption. Hatched areas represent the region of the ORF that is outside the homologous region. (B) *Integration-specific PCR analysis.* Genomic DNA was prepared from wild-type *P. berghei* and drug resistant parasite clones and PCR was performed using the primer pairs indicated in panel A. The presence of the 1.2 kb integration-specific PCR product (P3/P2) but not the 1.7 kb WT locus-specific PCR product (P1/P2) in the PbROM1(−) lanes confirm gene disruption. Note that WT lanes show the presence of the wild-type locus (P1/P2) as expected but not the integration locus (P3/P2). (C) *RT-PCR confirmation of PbROM1 disruption.* Salivary gland sporozoites from PbROM1(−)-infected mosquitoes did not express PbROM1, as expected. PbCS was used as a positive control and can be seen expressed in both WT and PbROM1(−) sporozoites.

To gain insights on PbROM1 function we disrupted the gene by homologous recombination and investigated the effects of gene loss on parasite development. Gene disruption was achieved by inserting a DNA fragment encoding a drug resistance marker into the open reading frame of PbROM1 (Figure 3A). Gene disruption was confirmed by insertion-specific PCR that identifies the disrupted locus from the wild-type locus (Figure 3B). In addition, disruption was confirmed by the absence of the transcript in PbROM1(−) sporozoites (Figure 3C).

### PbROM1 is required for efficient transition of ookinetes into oocysts but is not required for sporozoite invasion of salivary glands

**Figure 4:**
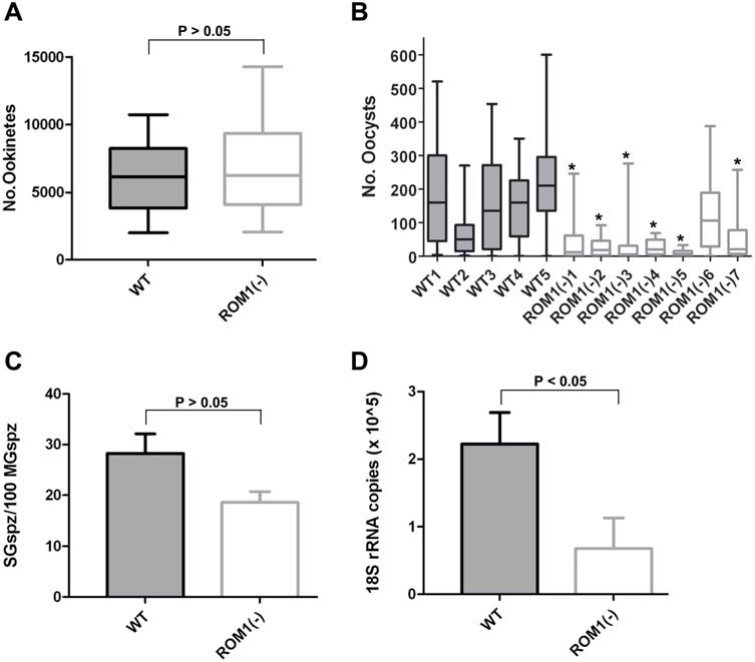
PbROM1 is required for efficient infection of mosquito midgut and mouse hepatocytes. (A) *PbROM1 is not required for ookinete formation.* Individual midguts from mosquitoes fed either on WT- or ROM1(−)-infected mice were analyzed for ookinete numbers. ROM1(−) parasites differentiated into ookinetes as efficiently as wild-type parasites (P>0.05, unpaired t test). (B) *PbROM1(−) ookinetes are impaired in the ability to form oocysts.* In six out of seven experiments, mosquitoes fed on mice infected with ROM1(−) parasites formed significantly fewer oocysts compared to four out of five experiments using mice infected with WT parasites. Experiments labeled ROM1(−)4, ROM1(−)5 and ROM1(−)6 were performed using an independent clone. Oocysts were counted on day 15 after blood feeding (*: P<0.05, ANOVA, Tukey's multiple comparison test). (C) *PbROM1 is not required for efficient invasion of mosquito salivary glands.* Sporozoites were isolated from midguts and salivary glands of mosquitoes (day 25–26) infected with WT and ROM1(−) parasites and counted on a hemocytometer. To estimate the efficiency of sporozoite infection of salivary glands, total midgut sporozoites were normalized for prevalence (mosquito infectivity) and mean oocysts per mosquito (day 15) for each experiment (P >0.05, unpaired t test). Bars show mean±SEM. (D) *ROM1 is required for efficient infection of the mouse liver.* The same number (1000) of WT or ROM1(−) salivary gland sporozoites were injected intravenously into mice and the efficiency of liver infection was measured 36 h later by quantitative PCR of *P. berghei* 18S rRNA normalized using mouse GAPDH (P<0.05, unpaired t test). Parasite load in livers of mice infected with mutant sporozoites was 68% lower than in livers infected by wild-type sporozoites.

We examined the possible function of PbROM1 in ookinetes by feeding PbROM1(−) parasites to mosquitoes. Ookinete efficiency of midgut invasion was assessed by counting the resulting number of oocysts. Disruption of the PbROM1 gene did not affect ookinete formation (Figure 4A and Table S1). However, in 6/7 experiments we found strong reduction in oocyst numbers (Figure 4B and Tables S2 and S3). These results suggest that loss of PbROM1 function impairs the ability of ookinetes to form oocysts. Subsequent development of PbROM1(−) parasites appears to be normal. The number of sporozoites formed by PbROM1(−) oocysts was similar to wild-type oocysts and no differences of salivary gland invasion could be detected (Figure 4C and Table S4). This result is consistent with the apparent lack of ROM1 protein expression in midgut sporozoites (Figure 1B).

### PbROM1(−) sporozoites infect the liver less efficiently

To investigate whether PbROM1 plays a role in liver infection, we injected mice intravenously with an equal number of WT and PbROM1(−) sporozoites. The efficiency of infection was dose dependent and mice infected with PbROM1(−) parasites showed a consistent delay in the pre-patent period by one day or more compared to mice infected with wild-type sporozoites (Table 1). Efficiency of infection was also assessed by quantifying parasite loads in livers infected with equal numbers of mutant or wild-type sporozoites. Livers of mice infected with the mutant sporozoite had a 68% lower parasite load compared with mice infected with wild-type sporozoites (Figure 4D). This suggests that PbROM1 is required for efficient hepatocyte infection.

**Table 1:** Prepatent period of blood infection is longer for mice infected with PbROM1(−) sporozoites

Parasite population	Rate of infection	Prepatent period
WT-150	7/8	5.1
WT-1000	7/7	4.8
WT-10000	3/3	3.3
ROM1(−)-150	0/8	
ROM1(−)-1000	6/8	5.7
ROM1(−)-10000	6/6	4.8

Mice were injected intravenously with the indicated number of wild-type (WT) or PbROM1(−) salivary gland sporozoites. Rate of infection is expressed as the number of mice infected/total number of mice injected with sporozoites. Pre-patent period is the number of days between sporozoite injection and the appearance of blood stage parasites upon examination of at least 25,000 RBCs.

### PbROM1 is not required for gliding motility

**Figure 5:**
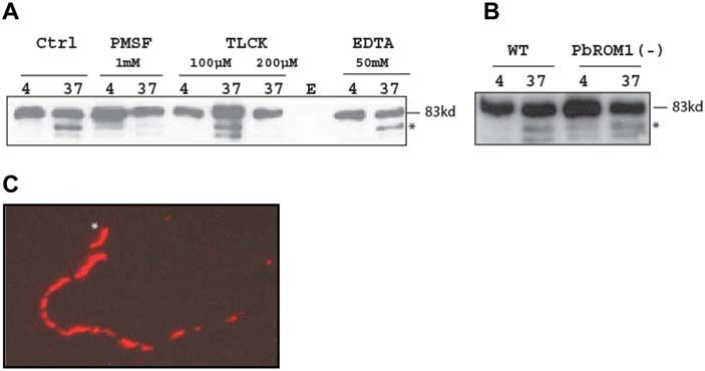
PbTRAP is not a substrate for PbROM1. (A) *PbTRAP is cleaved by a serine protease.* Total cell lysate from 3×10^4^ salivary gland sporozoites was loaded in each lane of a 4–25% denaturing SDS-PAGE. Western blot was performed using an anti-PbTRAP-repeat rabbit polyclonal antibody. The TRAP fragment recognized by the anti-repeat antibody but not by antibody against the cytoplasmic tail is indicated with an asterisk. Ctrl, control; E, empty lane. (B) *PbTRAP processing in PbROM1(−) sporozoites.* Experiments with 3×10^4^ sporozoites/lane were conducted as described for experiments in panel A with wild-type (WT) and mutant parasites. (C) *Gliding motility of PbROM1(−) sporozoites.* PbROM1(−) salivary gland sporozoites were placed in a 2-well chamber slide coated with BSA and incubated at 37°C for 30 min. After fixation with paraformaldehyde CSP trails were detected with an anti-CSP antibody (3D11) and a rhodamine-conjugated anti-mouse secondary antibody. *, sporozoite at the leading end of the trail.

To determine if the defect observed in hepatocyte infection is due to a defect in motility, we performed a sporozoite gliding assay. PbROM1(−) sporozoites are motile as observed by circumsporozoite protein trails on glass slides (Figure 5C). PbTRAP, the parasite adhesin essential for gliding motility [41], is proteolytically processed by a serine protease (Figure 5A [42],[43]). This processing appears to occur independent of ROM1 (Figure 5B). This suggests that the reduction in parasite numbers may not be due to impairment in motility but rather a defect in invasion and/or a subsequent defect in development.

### PbROM1 disruptant parasites are impaired in blood stage infection

**Figure 6:**
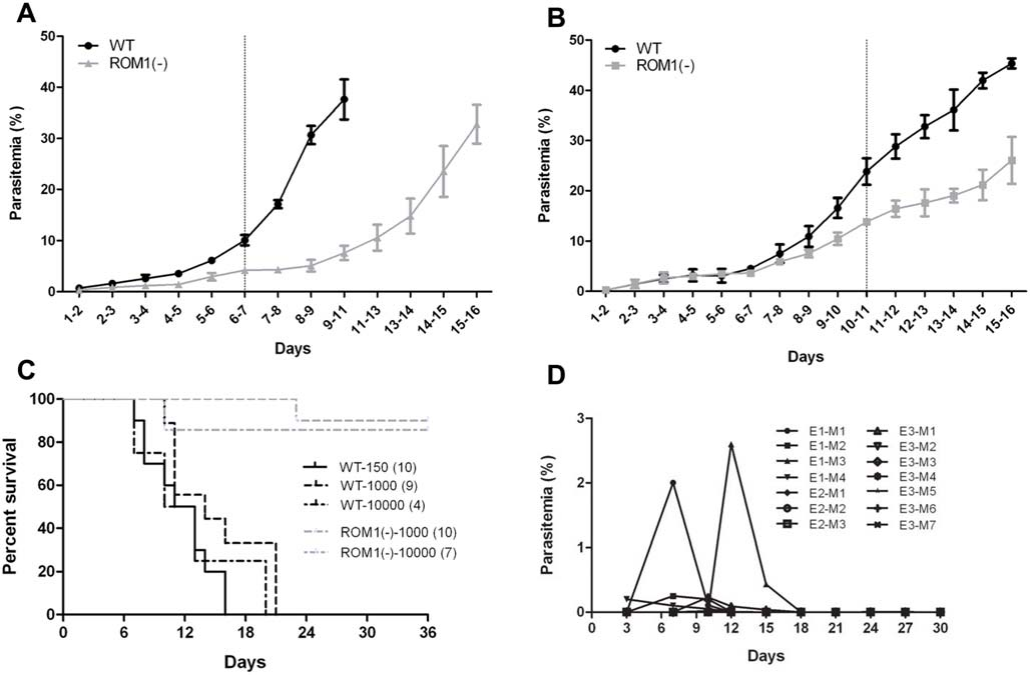
PbROM1(−) blood-stage parasites grow slower. Mice infected with the mutant parasite survive longer and become protected from a WT parasite challenge. Swiss Webster mice were injected intravenously with 1,000 wild-type (WT) or PbROM1(−) salivary gland sporozoites (Spz) (A) or intraperitonealy with 10^5^ infected RBCs (iRBC) (independent clone (B)) and parasitemia was measured daily and expressed as mean of two consecutive days. Three to four mice were used for each group and parasitemia is expressed as mean±SEM. The dotted line represents the point beyond which parasitemia in WT and ROM1(−) differed significantly (P<0.05, repeated measures ANOVA). (C) *Survival of mice infected with either wild-type (WT) or PbROM1(−) sporozoites.* Swiss Webster mice were injected *intravenously* with the indicated number of either WT or PbROM1(−) sporozoites and animal survival was monitored daily. PbROM1(−) infected mice survive significantly longer than WT infected mice (P<0.0001). Numbers in parenthesis indicate the number of mice assayed. (D) *Mice that clear PbROM1(−) infection are protected from WT parasite challenge.* Mice (M) from three experiments (E) such as the ones described in panel A and B that had survived PbROM1(−) infection and cleared the parasites were re-infected by either a) intravenous (*i.v*) injection of 10^4^ wild-type sporozoites (E1), b) intra-peritoneal injection of 10^6^ infected RBCs (E2), or c) intra-venous injection of 10^6^ infected RBCs (E3) at least 30 days after the clearance of the original PbROM1(−) infection. Seven out of 14 mice developed very mild parasitemia (0.004%–2.6%) that was subsequently completely cleared. These mice were also protected from a subsequent 2^nd^ and a 3^rd^ WT parasite challenge (Table 2).

Parasitemia develops slower in animals infected with PbROM1(−) parasites compared to WT infected animals (Figure 6A and 6B). This phenotype is observed in animals infected by injection of sporozoites (Figure 6A) as well as when bypassing liver invasion by injecting infected RBCs (iRBCs) (Figure 6B). This slow-growth phenotype is specific to PbROM1 disruptants as another rhomboid (ROM3) disruptant and an oocyst capsule protein disruptant [44] have growth kinetics similar to wild-type parasites (data not shown).

### PbROM1(−)-infected mice have greatly enhanced lifespan

Mice infected with PbROM1(−) parasites survive better than those infected with WT parasites (Figure 6C). Animals infected with PbROM1(−) parasites reach peak parasitemia of >35%, similar to WT parasites. At such high parasitemia, animals infected with WT parasites succumb to the infection. On the other hand, more than 80% of animals infected with PbROM1(−) parasites survive and eventually clear the parasites from their blood stream.

### PbROM1(−) infection protects mice from challenge with a lethal dose of wild-type parasites

Mice that had cleared PbROM1(−) parasites from their bloodstream were challenged by intravenous injection of 10^5^ WT iRBCs at least 30 d after the last circulating parasite was detected. Peak parasitemia in 12/14 mice after WT challenge ranged between 0.004%–2.6% (Figure 6D). Importantly, all the animals were able to successfully clear the wild-type parasites (Table 2). This protective immunity lasts for at least 7–9 months after the initial PbROM1(−) parasite exposure (Table 2). It is possible that the reduced RBC invasion efficiency of PbROM1(−) merozoites may trigger this protective immune response.

**Table 2:** PbROM1(−) parasites generate protective immunity

Expt	PbROM1(−) infection	No. parasites	1^st^ challenge (10^5^)		2^nd^ challenge (10^5^)		3^rd^ challenge (10^5^)	
			Infected	Protected	Infected	Protected	Infected	Protected
1	Sporozoite	10^4^	3/4 (30d)	4/4 (30d)	4/4 (60d)	4/4 (60d)	4/4 (9m)	4/4 (9m)
2	iRBC (*i.p*)	10^6^	1/3 (30d)	3/3 (30d)	3/3 (60d)	3/3 (60d)	3/3 (9m)	3/3 (9m)
3	iRBC (*i.p*)	10^6^	3/7 (30d)	7/7 (30d)	5/7 (7m)	7/7 (7m)		

Swiss Webster mice were infected with PbROM1(−) either by intravenous injection of sporozoites or intravenous or intraperitoneal (*i.v* or *i.p*) administration of iRBCs (cf. Figure 5D). Animals that cleared PbROM1(−) infection (Figure 5D) were used for wild-type parasite challenges at least 30 days after the last observed parasite. PbROM1(−) parasite clearance was confirmed by transfer of 3×10^7^ RBCs from all parasite-free animals to naïve mice. None became infected. These mice were then challenged *intravenously* with 10^5^ wild-type *P. berghei* iRBCs and monitored for infection. All the animals were protected (1^st^ challenge). These animals were also protected from a second and third challenge. Numbers in parenthesis indicates the number of days (d) or months (m) after the last PbROM1(−) parasite was observed. Infected, number of animals that had blood stage parasites; Protected, number of animals that cleared the blood-stage infection.

## Discussion

Invasion requires the specific recognition and attachment of parasite surface ligands to host cell receptors and subsequent processing of the bound ligands to facilitate detachment and entry into the host cell. This can be achieved by proteolytic processing of protein ectodomains [19] or in some cases by processing within the protein's transmembrane domain [18]. *Plasmodium* AMA1, EBL, RBL and TRAP proteins function in host-cell interaction and all have potential rhomboid cleavage sites within their predicted transmembrane domains. Recent studies using an *in vitro* mammalian cell-based assay indicate that *Plasmodium* ROM1 and ROM4 are able to cleave AMA1, EBL, RBL and TRAP members within their membrane-spanning domains [26],[28]. This suggests an important function for rhomboid proteins in invasion of host cells. In the present study we undertook a genetic approach to investigate the role of *Plasmodium berghei* rhomboid 1 (PbROM1) during the parasite development in the mammalian host and the mosquito vector.

Microarray analysis of *P. falciparum* genes identified PfROM1 as being expressed in both the mosquito and the asexual forms of the parasite [39]. Similarly, *P. berghei* ROM1 is also expressed in the mosquito and in its mammalian host [29],[40]. In agreement with the mRNA expression data, we find PbROM1 protein to be expressed in schizonts, in free merozoites and in sporozoites after salivary gland invasion. Though PbROM1 transcripts can be found in ookinetes (Figure S2), we could not detect the protein by indirect immunofluorescence. This may be due to the low abundance of the protein in this parasite form. The difference in PbROM1 protein expression between midgut and salivary gland sporozoites suggests post-transcriptional gene regulation. Incompletely spliced PbROM1 transcripts can be found in mature oocysts and sporozoites isolated from these oocysts (Figure S2). Furthermore, the ROM1 mRNA may be translationally regulated. Post-transcriptional regulation has been observed for a number of genes, especially in the sexual stages and plays an important role in *Plasmodium* development [29],[45],[46].

Our genetic analysis indicates that PbROM1 functions in both the vertebrate and mosquito stages. This is based on the observation that PbROM1(−) ookinetes form fewer oocysts, sporozoites isolated from infected mosquitoes infect the mouse liver less efficiently and the growth kinetics of the asexual forms is significantly delayed. Hence the phenotype of PbROM1(−) parasites points to ROM1 roles during cell invasion. However, a role in intracellular development cannot be formally excluded. We believe this to be less likely for several reasons. First, the mutant parasites fully complete development after invasion of the mosquito midgut epithelium, mouse liver and mouse RBCs. Second, WT and ROM1(−) ookinetes (Table S1), sporozoites (Table S2) and blood-stage merozoites (data not shown) develop equally well. Third, the ROM1 protein localizes to merozoite and sporozoite micronemes (an organelle that secrete proteins involved in invasion), in addition to the sporozoite surface. Together, these observations point to a role for ROM1 in host cell invasion.

Mice infected with PbROM1(−) parasites survive longer and are able to clear the infection efficiently. Those that clear the infection develop long-lasting immunity against a subsequent lethal wild-type *P. berghei* challenge. The immunity developed by PbROM1(−)-infected mice could be a result of slower infection, which provides the animal with an opportunity to mount a better immune response. Another interesting possibility is that parasite proteins normally processed by PbROM1 during invasion modulate the immune response. The absence or reduced levels of these cleaved proteins would allow the animals to develop immunity against the parasite. Interestingly, the *Toxoplasma gondii* ROM1 orthologue has also been shown to be required for efficient growth and invasion of host cells [47]. In addition to its role in invasion, TgROM1 also appears to play a role in intracellular replication as they form fewer parasites within the parasitophorous vacuole [47]. However, PbROM1 does not appear to play a significant role in the development neither of sporozoites within oocysts (Table S4) nor of merozoites within schizonts (data not shown). However, a role for PbROM1 in parasite replication in the mouse liver cannot be excluded. The observed differences between *Plasmodium* and *Toxoplasma* could represent a species-specific difference of ROM1 function.

Even though PbROM1(−) parasites are defective in multiple invasive stages, they do complete their life cycle successfully in both the vertebrate and invertebrate hosts. It is possible that in PbROM1(−) parasites, impairment of proteolytic processing only delays parasite invasion. Alternatively or in addition, other rhomboid proteins and/or proteases may take over the function of PbROM1, albeit with lower efficiency. There is precedent for such redundant function from i*n vitro* data suggesting that some substrates are cleaved well by either PfROM1 or PfROM4, while other substrates are cleaved by both enzymes, albeit at different efficiencies [26]. A number of candidate substrates for PbROM1 such as AMA1 have been identified using mammalian cell-based assays [26]. However, these would have to be validated by *in vivo* experiments and factors such as spatial and temporal regulation of the protease and its substrate(s) are also expected to play a role. Our results suggest that PbTRAP, the parasite adhesin required for sporozoite motility, is cleaved by a serine protease. The protease inhibitors used does not necessarily inhibit only TRAP processing, but would be expected to inhibit several other serine proteases. However, the assay specifically measures only TRAP processing. TRAP is processed in the absence of ROM1 suggesting that it might not be a substrate. Alternatively, as discussed above, TRAP processing in ROM1(−) parasites could be due to functional redundancy. Data from *in vitro* processing assays suggest that this is unlikely because ROM4 but not ROM1 was able to cleave TRAP [26].

In conclusion, this study points to distinct roles for *Plasmodium berghei* ROM1 throughout parasite development. The lack of an effective vaccine is attributed to the high degree of antigenic variation [48] and the ability of the parasite to switch invasion pathways [49]–[52]. On the other hand, a common phenomenon in the different invasion pathways could be the need for processing and release of the adhesins. For instance, processing of EBA175 within the membrane domain is essential for invasion [28]. As suggested by our genetic analysis, targeting rhomboid proteins offers an attractive new approach to the control of malaria.

## Supporting Information

Figure S1 Conservation of catalytic residues between *Plasmodium* and *Drosophila* ROM1. The essential *Drosophila* Asparagine-Serine-Histidine catalytic triad (rhomboid motif), is conserved in *Plasmodium* ROM1 (asterisk). Other surrounding amino acids shown are also important for rhomboid protein function in *Drosophila*. The catalytic residues are predicted to be present within the transmembrane domains (shaded gray). *P. berghei*: *Plasmodium berghei*; Py: *Plasmodium yoelli*; Pf: *Plasmodium falciparum*; Dm: *Drosophila melanogaster*. The number of amino acids of each protein is indicated to the right.
(0.03 MB PDF)

Figure S2 Gene structure of PbROM1. The top diagram shows the canonical intron/exon structure of PbROM1 (exons in blue). EST sequences available from Genbank and PlasmoDB for gradient-purified ookinetes (yellow), sporozoites purified from either infected midguts (orange) or salivary glands (red) and from developing oocysts (brown) are shown below the PbROM1 structure. Genbank accession numbers are given alongside each EST. Incompletely spliced forms can be observed in the developing oocyst (day 10–12) and midgut sporozoites (CB603492 and DC216124).
(0.06 MB PDF)

Table S1 PbROM1 is not required for ookinete formation
(0.03 MB PDF)

Table S2 PbROM1 is required for efficient infection of the mosquito
(0.06 MB PDF)

Table S3 One way ANOVA test for assessing statistical significance of differences in WT and ROM1(−) oocyst numbers
(0.05 MB PDF)

Table S4 PbROM1 is not required for sporozoite invasion of mosquito salivary glands
(0.05 MB PDF)
