# Supplementary figures and images for: Distinct Roles of Plasmodium Rhomboid 1 in Parasite Development and Malaria Pathogenesis
Source: PLoS Pathog. 2009 Jan 16;5(1):e1000262. doi: 10.1371/journal.ppat.1000262 (PMC2607553; doi:10.1371/journal.ppat.1000262)

### Publicly owned structure

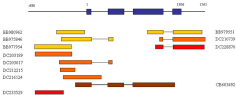

1991

Supplement: Figure S2 — Gene structure of PbROM1. The top diagram shows the canonical intron/exon structure of PbROM1 (exons in blue). EST sequences available from Genbank and PlasmoDB for gradient-purified ookinetes (yellow), sporozoites purified from either infected midguts (orange) or salivary glands (red) and from developing oocysts (brown) are shown below the PbROM1 structure. Genbank accession numbers are given alongside each EST. Incompletely spliced forms can be observed in the developing oocyst (day 10–12) and midgut sporozoites (CB603492 and DC216124). (0.06 MB PDF) [file ppat.1000262.s002.pdf]
