# Supplementary material for: Distinct Roles of Plasmodium Rhomboid 1 in Parasite Development and Malaria Pathogenesis
Source: PLoS Pathog. 2009 Jan 16;5(1):e1000262. doi: 10.1371/journal.ppat.1000262 (PMC2607553; doi:10.1371/journal.ppat.1000262)
Supplement: Table S1 — PbROM1 is not required for ookinete formation (0.03 MB PDF) [file ppat.1000262.s003.pdf]

Table S1

PbROM1 is not required for ookinete formation

Experiment	Ookinetes/mosquito	Prevalence (%)	P value
**1**. WT (5)	6592	100	
**2**. WT(5)	5697	100	
**1.** PbROM1(-) (10)	6917	100	
**2**. PbROM1(-) (10)	6811	100	
**Total**			
WT (10)	6144	100	
PbROM1(-) (20)	6864	100	> 0.05

The mean number of ookinetes/mosquito was determined by examination of Giemsa-stained smears of mosquito midgut contents as described in Materials and Methods. Numbers in parenthesis indicate the number of individual mosquitoes that were assayed for ookinete numbers.
