# Supplementary material for: Distinct Roles of Plasmodium Rhomboid 1 in Parasite Development and Malaria Pathogenesis
Source: PLoS Pathog. 2009 Jan 16;5(1):e1000262. doi: 10.1371/journal.ppat.1000262 (PMC2607553; doi:10.1371/journal.ppat.1000262)
Supplement: Table S2 — PbROM1 is required for efficient infection of the mosquito (0.06 MB PDF) [file ppat.1000262.s004.pdf]

Table S2

PbROM1 is required for efficient infection of the mosquito

	Oocysts/midgut		Prevalence (%)	Inhibition (%)	P value
	Mean	Median			
**Expt 1**					
WT (24)	167	135	87		
WT (23)	79	50	100		
WT (15)	182	160	100		
PbROM1(-) (23)	40	12	83	76 - 93	
PbROM1(-) (23)	28	18	91	65 - 89	
PbROM1(-) (16)	3	5	87	90 - 97	
**Expt 2**					
WT (16)	163	160	94		
WT (25)	213	210	97		
PbROM1(-) (24)	26	20	88	88 - 91	
PbROM1(-) (20)	10	7	85	96 - 97	
PbROM1(-) (25)	124	106	92	22 - 50	
PbROM1(-) (20)	55	21	90	87 - 90	
**Total**					
WT (103)	160	150	95		
PbROM1(-) (153)	47	15	89	90	< 0.0001

A total of 5 mice infected with WT parasites and 7 mice infected with ROM1(-) parasites were each fed to a different group of mosquitoes. Oocysts were counted 15 days after infection. Numbers in parenthesis indicate the number of individual mosquitoes that were examined. Inhibition of oocyst formation was calculated using median oocyst values. The range of inhibition observed based on the lowest and highest median oocyst numbers of WT infection is shown.
