# Supplementary material for: Distinct Roles of Plasmodium Rhomboid 1 in Parasite Development and Malaria Pathogenesis
Source: PLoS Pathog. 2009 Jan 16;5(1):e1000262. doi: 10.1371/journal.ppat.1000262 (PMC2607553; doi:10.1371/journal.ppat.1000262)
Supplement: Table S3 — One way ANOVA test for assessing statistical significance of differences in WT and ROM1(−) oocyst numbers (0.05 MB PDF) [file ppat.1000262.s005.pdf]

Table S3

One way ANOVA test for assessing statistical significance of differences in WT and ROM1(-) oocyst numbers

Mean Oocysts	Infection	182<br>WT1	79<br>WT2	167<br>WT3	162<br>WT4	213<br>WT5
182	WT1					
79	WT2	ns				
167	WT3	ns	ns			
162	WT4	ns	ns	ns		
213	WT5	ns	***	ns	ns	
40	ROM1(-)1	**	ns	***	**	***
28	ROM1(-)2	***	ns	***	**	***
30	ROM1(-)3	***	ns	***	**	***
26	ROM1(-)4	***	ns	***	***	***
10	ROM1(-)5	***	ns	***	***	***
124	ROM1(-)6	ns	ns	ns	ns	ns
55	ROM1(-)7	**	ns	**	*	***

An. stephensi mosquitoes were fed separately on one of 5 mice infected with WT parasites or on one of 7 mice infected with ROM1(-) parasites. In six out of seven experiments, mosquitoes that were fed on ROM1(-) parasites formed significantly lower number of oocysts when compared to four out of five experiments using mice infected with WT parasites. P values and confidence intervals are corrected for multiple comparisons using the Boferroni’s multiples comparison test (***: P<0.001; **: P<0.01; *: P<0.05, ns: P>0.05)
