# Supplementary material for: Distinct Roles of Plasmodium Rhomboid 1 in Parasite Development and Malaria Pathogenesis
Source: PLoS Pathog. 2009 Jan 16;5(1):e1000262. doi: 10.1371/journal.ppat.1000262 (PMC2607553; doi:10.1371/journal.ppat.1000262)
Supplement: Figure S1 — Conservation of catalytic residues between Plasmodium and Drosophila ROM1. The essential Drosophila Asparagine-Serine-Histidine catalytic triad (rhomboid motif), is conserved in Plasmodium ROM1 (asterisk). Other surrounding amino acids shown are also important for rhomboid protein function in Drosophila. The catalytic residues are predicted to be present within the transmembrane domains (shaded gray). P. berghei: Plasmodium berghei; Py: Plasmodium yoelli; Pf: Plasmodium falciparum; Dm: Drosophila melanogaster. The number of amino acids of each protein is indicated to the right. (0.03 MB PDF) [file ppat.1000262.s001.pdf]

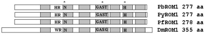
HR N
GAST
H
PbROM1 277 aa
HR N
GAST
H
PyROM1 277 aa
HR N
GAST
H
PfROM1 278 aa
WR N
GASG
H
DmROM1 355 aa

Fig S1
