# Supplementary material for: Distinct Roles of Plasmodium Rhomboid 1 in Parasite Development and Malaria Pathogenesis
Source: PLoS Pathog. 2009 Jan 16;5(1):e1000262. doi: 10.1371/journal.ppat.1000262 (PMC2607553; doi:10.1371/journal.ppat.1000262)
Supplement: Table S4 — PbROM1 is not required for sporozoite invasion of mosquito salivary glands (0.05 MB PDF) [file ppat.1000262.s006.pdf]

Table S4

PbROM1 is not required for sporozoite invasion of mosquito salivary glands

Experiment	Oocysts/mosquito	Spz/oocyst	SG spz/ 100 MG spz	Prevalence (%)	P value
**1.** WT (19)	213	723	29	96	
**2.** WT (12)	163	939	21	100	
**3.** WT (15)	182	842	34	100	
**1.** PbROM1(-) (17)	26	1013	23	87.5	
**2.** PbROM1(-) (15)	55	285	18	90	
**3.** PbROM1(-) (18)	124	973	13	92	
**4.** PbROM1(-) (14)	10	2214	21	85	
**Total**					
WT (46)	186	835	25		
PbROM1(-) (54)	54	1121	20		> 0.05

Sporozoites were isolated either from midgut oocysts (day 25-26) or from salivary glands (day 25-26) of infected mosquitoes and counted with the aid of a hemocytometer. Sporozoite counts were normalized against total oocyst count (day 15) and mosquito infection prevalence. As all salivary gland sporozoites come from oocysts, the sum of midgut and salivary gland sporozoites was taken as total oocyst sporozoites. Numbers in parenthesis indicate the number of individual mosquitoes that were analyzed for sporozoites.
